# Increased IKKϵ protein stability ensures efficient type I interferon responses in conditions of TBK1 deficiency

**DOI:** 10.3389/fimmu.2023.1073608

**Published:** 2023-03-03

**Authors:** Julia Wegner, Charlotte Hunkler, Katrin Ciupka, Gunther Hartmann, Martin Schlee

**Affiliations:** Institute of Clinical Chemistry and Clinical Pharmacology, University Hospital Bonn, Bonn, Germany

**Keywords:** innate immunity, pathogens, virus infection, nucleic acid sensing, cGAS, TBK1, IKKϵ, protein degradation

## Abstract

TBK1 and IKKϵ are related, crucial kinases in antiviral immune signaling pathways downstream of cytosolic nucleic acid receptors such as cGAS and RIG-I-like receptors. Upon activation, they phosphorylate the transcription factors IRF3 and IRF7 and thereby initiate the expression of type I interferons and antiviral effectors. While point mutation-induced loss of TBK1 kinase activity results in clinical hyper-susceptibility to viral infections, a complete lack of TBK1 expression in humans is unexpectedly not associated with diminished antiviral responses. Here, we provide a mechanistic explanation for these so-far unexplained observations by showing that TBK1 controls the protein expression of its related kinase IKKϵ in human myeloid cells. Mechanistically, TBK1 constitutively diminishes the protein stability of IKKϵ independent of TBK1 kinase activity but dependent on its interaction with the scaffold protein TANK. In consequence, depletion of TBK1 protein but not mutation-induced kinase deficiency induces the upregulation of IKKϵ. Due to the functional redundancy of the kinases in cGAS-STING and RIG-I-like receptor signaling in human myeloid cells, enhanced IKKϵ expression can compensate for the loss of TBK1. We show that IKKϵ upregulation is crucial to ensure unmitigated type I interferon production in conditions of TBK1 deficiency: While the type I interferon response to *Listeria monocytogenes* infection is maintained upon TBK1 loss, it is strongly diminished in cells harboring a kinase-deficient TBK1 variant, in which IKKϵ is not upregulated. Many pathogens induce TBK1 degradation, suggesting that loss of TBK1-mediated destabilization of IKKϵ is a critical backup mechanism to prevent diminished interferon responses upon TBK1 depletion.

## 1 Introduction

TANK-binding kinase (TBK1) plays a crucial role in the host defense against pathogens by functioning as a signaling intermediate downstream of cytosolic or endosomal nucleic acid-sensing pattern recognition receptors (PRRs). Upon PRR activation, TBK1 promotes the C-terminal phosphorylation and activation of the transcription factors interferon-regulatory factor 3 (IRF3) and IRF7 (1, 2). They induce the expression of type I interferon (IFN) and antiviral effector genes, which are essential for combating infections. The most relevant antiviral type I IFN-inducing pathways in human immune cells signal through the adaptor proteins stimulator of IFN genes (STING), mitochondrial antiviral signaling protein (MAVS), and TIR domain-containing adaptor inducing IFNβ (TRIF), which integrate signals from PRRs (3). Specifically, STING functions downstream of the cytosolic DNA sensor cyclic guanosine monophosphate-adenosine monophosphate (cGAMP) synthase (cGAS), MAVS downstream of the cytosolic RNA receptors retinoic acid-inducible gene I (RIG-I) and melanoma differentiation-associated gene 5 (MDA5), and TRIF binds to the endosomal toll-like receptor 3 (TLR3). Upon receptor activation, all three adaptor proteins recruit and activate TBK1 (4). Besides TBK1, IκB kinase ϵ (IKKϵ) has also been implicated in controlling type I IFN responses downstream of PRRs. Sharing a sequence homology of more than 60%, TBK1 and IKKϵ are structurally and functionally closely related (5). Still, TBK1 was found more important than IKKϵ in type I IFN induction in mice (6, 7), which might be at least partially related to the distinct expression patterns of the kinases: While TBK1 is constitutively and ubiquitously expressed, IKKϵ expression in mice is largely restricted to immune cells but can be induced in somatic cells by pro-inflammatory cytokines (8, 9).

Phosphorylation of IRF3 and IRF7 by TBK1 and IKKϵ (TBK1/IKKϵ) requires *trans*-autophosphorylation at serine 172 in the activation loop of the kinases (10). Moreover, it depends on the assembly of TBK1/IKKϵ complexes with the scaffold proteins TRAF family member-associated NF-κB activator (TANK), NF-κB-activating kinase-associated protein 1 (NAP1), and Similar to NAP TBK1 adaptor (SINTBAD) (11–15). Interaction with these scaffold proteins is mediated by the coiled-coil domain 2 (CCD2) in the C-terminal regions of TBK1 (16). TANK, NAP1, and SINTBAD seem to bind TBK1 in a mutually exclusive manner, resulting in the formation of alternative TBK1 complexes with potential distinct functions in nucleic acid receptor signaling and other cellular processes such as selective autophagy (16, 17).

Because of the elementary function of TBK1/IKKϵ in antiviral immunity, many viruses have evolved to block their activity. For example, TBK1 has been shown to be degraded by severe acute respiratory syndrome coronavirus 2 (SARS-CoV-2), herpes simplex virus 1 (HSV-1), and human immunodeficiency virus 1 (HIV-1) (18–21). Moreover, bacterial infection can also lead to TBK1 degradation, as shown for *Shigella* species (22).

While homozygous deletion of TBK1 in mice is embryonically lethal, a recent study describes human individuals with complete lack of TBK1 expression (23, 24). Surprisingly, these patients show a sufficient type I IFN response and do not suffer from recurrent infections (24). In contrast, humans harboring point mutations in TBK1 that result in a loss of its kinase activity are highly susceptible to severe viral infection (25). Until now, it has remained unclear why a complete loss of TBK1 expression is better tolerated in terms of immunocompetence than a mutation of TBK1 affecting its kinase function.

In this study, we provide a mechanistic explanation for the observed discrepancy. We show that in human immune cells, TBK1 continuously induces the degradation of its related kinase IKKϵ, independent of its enzymatic activity. In consequence, loss of TBK1 expression but not mutation-induced abolishment of TBK1 kinase activity causes the upregulation of IKKϵ. Since TBK1 and IKKϵ redundantly mediate IRF activation downstream of cGAS-STING and RIG-I-like receptors (RLRs), as demonstrated here, enhanced amounts of IKKϵ protein can compensate for the loss of TBK1. We show that in conditions of TBK1 deficiency, IKKϵ induction is crucial to ensure unmitigated type I IFN responses after specific activation of cGAS and RLRs, and also during infection with *Listeria monocytogenes*. Overall, our data provide evidence that human immune cells are equipped with an important backup mechanism that ensures effective antiviral and antibacterial responses even in case of pathogen-induced TBK1 degradation or genetic loss of TBK1.

## 2 Materials and methods

### 2.1 Cell culture

THP1-Dual suspension cells (InvivoGen) were cultivated in RPMI-1640 medium supplemented with 10% FCS, 100 U/ml penicillin, and 100 µg/ml streptomycin (complete RPMI). Adherent HEK293FT cells (Invitrogen) were cultivated in DMEM medium supplemented with 10% FCS, 100 U/ml penicillin, and 100 µg/ml streptomycin (complete DMEM).

### 2.2 Generation of knockout cell lines by CRISPR-Cas9 genome editing

The Alt-R CRISPR-Cas9 system from Integrated DNA Technologies was utilized to edit genes of interest, resulting in loss of protein expression from these genes. Different protocols were used for THP1-Dual cells and HEK293FT cells.

For THP1-Dual suspension cells, 0.4 µl of both gene-specific crRNA and tracrRNA (both 100 µM, see **Table 1**) were mixed, heat-denatured at 95°C for 5 min, and slowly cooled to room temperature (RT) to generate an RNA duplex serving as guideRNA. For double KOs, 0.2 µl of each crRNA was annealed with 0.4 µl tracrRNA. The RNA duplexes were then incubated for 20 min at RT with 1 volume of 36 µM Cas9 enzyme to facilitate complex formation. After washing THP1-Dual cells (5×10^5^ per electroporation reaction) once with PBS, they were resuspended in buffer R (Neon Transfection System kit, Thermo Fisher Scientific, 13.5 µl per electroporation reaction). 1.5 µl of the crRNA:tracrRNA: Cas9 complex and 3 µl of electroporation enhancer (10.8 µM) were added to the cell suspension and the complex was delivered into the cells using the Neon Transfection System device (Thermo Fisher Scientific, 1600 V, 3 pulses, 10 ms pulse width). The cells were transferred to prewarmed complete RPMI and were allowed to recover and expand for 3 days before single cell cloning was performed by limiting dilution seeding.

**Table 1:** Sequences of crRNAs used for CRISPR-Cas9 genome editing.

Gene	ID	Sequence (shown 5’ to 3’)	Genome editing of
TBK1	B	ATTCCTACGAGGCCCTTCAA	HEK293FT, MDMs
TBK1	C	TTTGAACATCCACTGGACGA	THP1-Dual, MDMs
TBK1	E	GAAGAACCTTCTAATGCCTA	MDMs
IKKϵ	C	GTTGCGGGCCTTGTACACAC	THP1-Dual

Sequences and design IDs of gene-specific crRNA predesigned by and ordered from Integrated DNA Technologies that were used for CRISPR-Cas9 genome editing of THP1-Dual cells, HEK293FT cells, and primary monocyte-derived macrophages (MDMs).

For HEK293FT cells, 0.3 µl of both TBK1-specific crRNA and tracrRNA (both 100 µM, see **Table 1**) were mixed with 29.4 µl nuclease-free duplex buffer and were then heated at 95°C for 5 min, followed by a slow cool-down to RT. After combining 9 µl of the formed RNA duplex with 9 µl of 2 µM Cas9 and 132 µl Opti-MEM, the mixture was incubated for 5 min at RT to allow complex formation. 7.2 µl Lipofectamine RNAiMAX (Thermo Fisher Scientific) diluted in Opti-MEM to a final volume of 150 µl were then combined with the RNA : Cas9 complex and incubated for 20 min at RT. In the meantime, HEK293FT cells (2.4×10^5^ per electroporation reaction) were washed once with PBS, detached from the cell culture flask, resuspended in 300 µl DMEM without antibiotics supplemented with 10% FCS, and were then combined with the transfection mixture in a 24-well plate. After 2 days of recovery, single cell cloning was performed.

After expansion, monoclonal cell lines were analyzed for loss of protein expression by SDS-PAGE and immunoblotting. Furthermore, genome editing was confirmed by Sanger sequencing of PCR-amplified genomic loci using TIDE (26). Genotypes are shown in **Table 2**.

**Table 2:** Genotypes of monoclonal KO cell lines.

	Insertions or deletions	
	*TBK1 locus*	*IKKϵ locus*
THP1-Dual (allele 1/allele 2)		
TBK1^-/-^ 1	-4/-4	
TBK1^-/-^ 2	-8/-8	
IKKϵ^-/-^ 1		-2/-2
IKKϵ^-/-^ 2		-2/-2
TBK1/IKKϵ^-/-^ 1	-8/-1	-8/-8
TBK1/IKKϵ^-/-^ 2	-2/-2	-2/-1
HEK293FT (allele 1/allele 2/allele 3)		
TBK1^-/-/-^ 1	-1/+1/+2	
TBK1^-/-/-^ 2	-8/-4/-1	

Insertions (+) and deletions (-) introduced by CRISPR-Cas9 genome editing at the respective loci. Due to the triploidy of chromosome 12 in HEK293FT cells, editing events at three TBK1 alleles are shown for these cells.

### 2.3 Primary human monocyte isolation

The studies involving primary human monocytes were approved by the local ethics committee (Ethikkommission der Medizinischen Fakultät Bonn) according to the ICH-GCP guidelines. Human peripheral blood mononuclear cells (PBMCs) were isolated from buffy coats of healthy, voluntary donors by Ficoll-Paque (Cytiva) density gradient centrifugation. Residual erythrocytes were removed by incubation in red blood cell lysis buffer (Roche). Isolation of primary monocytes from PBMCs was achieved by positive magnetic selection using CD14 microbeads (Miltenyi Biotec) according to the manufacturer’s instructions.

### 2.4 Differentiation of primary human monocytes to macrophages

Primary monocytes freshly isolated from human blood were differentiated to macrophages by cultivation in complete RPMI supplemented with 40 ng/ml M-CSF (Peprotech) at a cell density of 1.1×10^6^/ml in cell culture dishes. After three days, macrophages were detached by incubation for 10 min with ice-cold 5 mM EDTA/PBS, resuspended, and then used for CRISPR-Cas9 genome editing.

### 2.5 CRISPR-Cas9 genome editing of primary human monocyte-derived macrophages

To reduce the expression of TBK1 in primary monocyte-derived macrophages, polyclonal gene KOs were created. 0.6 µl of gene-specific crRNA and 0.6 µl tracrRNA (Integrated DNA Technologies, both 100 µM, see **Table 1**) were combined and were hybridized by incubation at 95°C for 5 min, followed by a slow cool down. Then, the RNA duplex was incubated with 1 volume of Cas9 (36 µM) for 20 min at RT. A non-targeting crRNA (Integrated DNA Technologies) was used as a negative control. After washing primary monocyte-derived macrophages (3.6×10^6^ per electroporation reaction) with PBS once, they were resuspended in buffer R (Neon electroporation kit, 21.6 µl per electroporation reaction) and combined with 2.4 µl of RNA: Cas9 complex and 4.8 µl electroporation enhancer (10.8 µM). Complex delivery into macrophages was achieved by electroporation using the Neon Transfection System device (Thermo Fisher Scientific, 1400 V, 2 pulses, 20 ms pulse width). Then, the cells were transferred to prewarmed RPMI supplemented with 10% FCS and 40 ng/ml M-CSF without antibiotics. After three days, the medium was exchanged to complete RPMI containing 40 ng/ml M-CSF and the cells were incubated for three further days before lysates were prepared for SDS-PAGE and immunoblot analysis, or qRT-PCR.

### 2.6 Stimulatory nucleic acids

High molecular weight polyinosinic-polycytidylic acid (polyI:C) was obtained from InvivoGen. Hybridization of G3-YSD (**Table 3**) was achieved by heating the combined single-stranded DNA oligonucleotides in NEB2 buffer (New England Biolabs) at 95°C for 5 min and subsequent cooling to RT at a rate of 1°C/min. Plasmid DNA (pDNA) was isolated from pBluescript-transformed *E. coli* K12 using a PureLink HiPure Plasmid Filter Midiprep Kit (Life Technologies) according to the manufacturer’s instructions.

**Table 3:** Stimulatory nucleic acids.

Name	Sequence (5’ to 3’)
G3-YSD, sense	GGGAAACTCCAGCAGGACCATTAGGG
G3-YSD, antisense	GGGTAATGGTCCTGCTGGAGTTTGGG

G3-YSD oligonucleotides used for direct stimulation of cells were ordered from Integrated DNA Technologies.

### 2.7 Cell stimulation

THP1-Dual cells (6×10^4^/well of a 96-well plate, 3.6×10^5^/well of a 24-well plate, or 7.2×10^5^/well of a 12-well plate) were seeded in 150 µl, 450 µl, or 900 µl complete RPMI for type I IFN reporter assay, qRT-PCR, or immunoblotting readout, respectively, and were stimulated with RIG-I/MDA5 (polyI:C) or cGAS (pDNA, G3-YSD) agonists. For type I IFN reporter assays, cells were stimulated in duplicates. Stimuli were complexed with the vehicle Lipofectamine 2000 (Invitrogen) according to the manufacturer’s instructions and were then added to the cells, resulting in a final volume of 200 µl (96-well plate), 1200 µl (24-well plate), or 2400 µl (12-well plate) with stimuli concentrations ranging from 10 ng/ml to 500 ng/ml, as indicated in the figure legends. IFNα2a (Miltenyi Biotec) was used as a control for equal IFN-stimulated response element (ISRE) reporter responsiveness of the different cell lines.

### 2.8 Plasmids

For lentiviral vector production and reconstitution of TBK1-deficient THP1-Dual cells, human TBK1 variants (WT and mutants) with C-terminal Flag tag were cloned into a pLVX-puro-EF1α vector *via* Gibson assembly using the primers listed in **Table 4**. Similarly, Flag-tagged human IKKϵ was cloned into the same vector for IKKϵ overexpression (see **Table 4**). For identification of IKKϵ ubiquitination by immunoprecipitation, GFP-tagged human IKKϵ was cloned into a pLVX-puro-EF1α vector *via* Gibson assembly (see **Table 4**).

**Table 4:** Cloning primers.

Name	Sequence (5’ to 3’)	Used for
IKKϵ GA fwd	ATTTCGACCCGGATCCGCGGCCGCG CCACCATGCAGAGCAC	Cloning of pLVX-puro-EF1α-IKKϵ-Flag
pLVX Flag GA rev	CCCCTACCCGGTAGAATTCACGCGT TCACTTATCGTCGTCATCCTTGTAA TC	Cloning of pLVX vectors encoding for C-terminally Flag-tagged proteins
pLVX IKKϵ-GFP IKKϵ fwd	TGAGGAATTTCGACCCGGATCC	Cloning of pLVX-puro-EF1α-IKKϵ-GFP
pLVX IKKϵ-GFP IKKϵ rev	TGACGATCCTTCCTTCAATGGATCC GACATCAGGAGGTGCTGGGA	
pLVX IKKϵ-GFP GFP fwd	TCCCAGCACCTCCTGATGTCGGATC CATTGAAGGAAGGATCGTCA	
pLVX IKKϵ-GFP GFP rev	TCCCCTACCCGGTAGAATTCACGCG TTTACTTGTACAGCTCGTCCATGC	
TBK1 muta fwd1	CTCCTTGGAATTTGCCCTTTTTGAGT	Cloning of TBK1 K38A-Flag
TBK1 muta fwd2	GAATTTCGACCCGGATCCGC	Cloning of pLVX vectors encoding for C-terminally Flag-tagged TBK1 with point mutations
TBK1 muta rev1	CTACCCGGTAGAATTCACGCG	
TBK1 C426A fwd	TGTGTTATGCCGCCAGAATTGCCAG	Cloning of pLVX-puro-EF1α-TBK1 C426A/C605A-Flag
TBK1 C426A rev	CTGGCAATTCTGGCGGCATAACACA	
TBK1 C605A fwd	CGCACTTTACAGATGAAGCCGTTAA AAAGT	
TBK1 C605A rev	ACTTTTTAACGGCTTCATCTGTAAA GTGCG	
TBK1 K38A fwd	GGTGATTTATTTGCTATCGCCGTAT TTAATAACATAAGC	Cloning of pLVX-puro-EF1α-TBK1 K38A-Flag
TBK1 K38A rev	GCTTATGTTATTAAATACGGCGATA GCAAATAAATCACC	
TBK1 L693A fwd	GGTATGAAGAAAGCCAAGGAAGAG ATGG	Cloning of pLVX-puro-EF1α-TBK1 L693A-Flag
TBK1 L693A rev	CCATCTCTTCCTTGGCTTTCTTCATA CC	
TBK1 K694E fwd	GTATGAAGAAATTAGAAGAAGAGA TGGAAGGGG	Cloning of pLVX-puro-EF1α-TBK1 K694E-Flag
TBK1 K694E rev	CCCCTTCCATCTCTTCTTCTAATTTC TTCATAC	
TBK1 L704A fwd	GGGTGGTTAAAGAAGCCGCTGAAAA TAACC	Cloning of pLVX-puro-EF1α-TBK1 L704A-Flag
TBK1 L704A rev	GGTTATTTTCAGCGGCTTCTTTAAC CACCC	
TBK1 S172A fwd	GAGCAGTTTGTTGCCCTGTATGGCAC	Cloning of pLVX-puro-EF1α-TBK1 S172A-Flag
TBK1 S172A rev	GTGCCATACAGGGCAACAAACTGCTC	
TBK1 pLVX GA fwd	GAATTTCGACCCGGATCCGCGGCCG CGCCACCATGCAGAGCACTTCTAAT CATCTGTG	Cloning of pLVX-puro-EF1α-TBK1-Flag

Primers used for cloning of TBK1 and IKKϵ plasmids. All oligonucleotides were ordered from Integrated DNA Technologies.

### 2.9 Lentiviral transduction and selection of cells

Lentiviral vectors were produced by transfection of HEK293FT cells with lentiviral transfer plasmid (1.6 µg of pLVX-puro-EF1α-TBK1-Flag (WT or mutant), pLVX-puro-EF1α-IKKϵ-Flag, pLVX-puro-EF1α-GFP, or pLVX-puro-EF1α-IKKϵ-GFP) and packaging plasmids (0.4 µg, 0.6 µg, and 1.1 µg of pRSV-rev, pMD2.G, and pMDL.g, respectively) using calcium phosphate transfection. Virus-containing cell supernatants were harvested 72 h post transfection. After resuspension of THP1-Dual cells in virus supernatant supplemented with polybrene (8 µg/ml), spin infection was performed for 1 h at 600×g and 32°C. 24 h post transduction, the medium was replaced with puromycin-containing medium (1 µg/ml) and selection of transduced cells was performed for 3 days.

### 2.10 *L. monocytogenes* infection

*L. monocytogenes* (wildtype strain EGD) was cultivated in BHI medium until reaching the log phase and was then stored at -80°C until infection was performed. THP1-Dual cells (3.6×10^5^/well of a 24-well plate) were washed with PBS twice and seeded in RPMI-1640 supplemented with 10% FCS but devoid of antibiotics. Infection with *L. monocytogenes* was performed in a final volume of 600 µl at a multiplicity of infection (MOI) of 1. After 2 h, cells were washed with antibiotic-free RPMI-1640 once and seeded in 600 µl RPMI-1640 supplemented with 10% FCS and 50 µg/ml gentamycin. After 24 h, the supernatant was harvested to determine type I IFN reporter activation and the cells were lysed for RNA isolation followed by qRT-PCR analysis.

### 2.11 Type I IFN reporter readout

Lucia luciferase secretion of THP1-Dual cells, controlled by an ISRE, was utilized as a surrogate parameter for IRF activation and type I IFN production. To determine type I IFN reporter activity, cell-free supernatant was harvested 16 or 24 h after stimulation, as indicated in the figure legend, and 30 µl supernatant were mixed with 30 µl coelenterazine solution (1 µg/ml in H_2_O) in a white 96-well F-bottom plate. Luciferase activity was determined immediately after coelenterazine addition with an EnVision 2104 Multilabel Reader device.

### 2.12 SDS-PAGE and immunoblotting

Cells were washed once with PBS, resuspended in RIPA lysis buffer supplemented with cOmplete Protease Inhibitor (Roche), and incubated on ice for 30 min. For the analysis of IRF3 phosphorylation, PhosSTOP phosphatase inhibitor cocktail (Roche) was additionally included in the lysis buffer. After clearing the lysates by centrifugation at 21,000×g for 10 min at 4°C, protein concentrations were determined using a Pierce BCA Protein Assay Kit (Thermo Fisher Scientific) according to the manufacturer’s instructions. 10–30 µg protein were loaded on an 8% SDS-PAGE gel and gel electrophoresis was performed for 90 min at 110 V. Proteins were transferred to a nitrocellulose membrane by blotting at 450 mA for 90 min. Total protein staining was performed by incubating the membrane for 5 min in Ponceau S solution (0.1% (w/v) Ponceau S in 5% acetic acid). After blocking the membranes with Intercept blocking buffer (LI-COR Biosciences), TBK1 and IKKϵ were detected with primary antibodies D1B4 and D20G4 (Cell Signaling Technology, 1:1000), respectively. Primary antibodies #2141, #8605 (both Cell Signaling Technology, 1:1000), and 15042-1-AP (Proteintech, 1:1000), were used to stain TANK, SINTBAD, and NAP1, respectively. Phosphorylation of IRF3 at S396 was detected with primary antibody 4D4G, while total IRF3 protein was stained with D6I4C (both Cell Signaling Technology, 1:1000). The housekeeping protein β-actin was detected with primary antibody 926-42212 (LI-COR Biosciences, 1:3000). Goat anti-rabbit IRDye800CW, goat anti-mouse IRDye800CW, and goat anti-mouse IRDye680 (LI-COR Biosciences, 1:10,000) were used as secondary antibodies. Blots were imaged using an Odyssey FC Dual imaging platform (LI-COR Biosciences). Quantification of signal intensities was performed using Image Studio Lite.

### 2.13 qRT-PCR

THP1-Dual cells or primary monocyte-derived macrophages were lysed in 350 µl RLT buffer (Qiagen) and were frozen at -80°C for at least 5 min. After thawing at RT, 1 volume of 70% ethanol was added and the sample was transferred to a Zymo III column (Zymo Research). After washing the column sequentially with 1 volume RW1 buffer (Qiagen) and 1 volume RNA wash buffer (Zymo Research), the column was dried by centrifugation for 2 min at maximum speed. The RNA was then eluted with RNase-free distilled H_2_O. DNase I digest was performed at 37°C for 30 min to destroy residual genomic DNA before the enzyme was inactivated at 70°C for 10 min in the presence of 2.5 mM EDTA. Reverse transcription was performed with RevertAid Reverse Transcriptase (Thermo Fisher Scientific) according to the manufacturer’s instructions using random hexamer primers (Integrated DNA Technologies). qRT-PCR reactions were performed in a QuantStudio 5 Real-Time PCR cycler (Thermo Fisher Scientific) using 5× EvaGreen QPCR Mix II (ROX) (Bio-Budget). Gene-specific primers used for quantification of mRNA levels (**Table 5**) were tested for efficiency by cDNA dilution.

**Table 5:** qRT-PCR primers.

Gene	Forward primer (5’ to 3’)	Reverse primer (5’ to 3’)
*IKBKE*	GAGCATTGGAGTGACCTTGTA	GATCCGGTACATGATCTCCTTG
*IFNB1*	CATTACCTGAAGGCCAAGGA	CAGCATCTGCTGGTTGAAGA
*IFIT1*	TCCACAAGACAGAATAGCCAGAT	GCTCCAGACTATCCTTGACCTG
*GAPDH*	AAGGTGAAGGTCGGAGTCAA	AATGAAGGGGTCATTGATGG

Primers used for mRNA expression analysis by qRT-PCR. All oligonucleotides were ordered from Integrated DNA Technologies.

### 2.14 Cycloheximide assay

THP1-Dual cells (3.6×10^5^ per well of a 24-well plate) were seeded in 450 µl complete RPMI. Then, 150 µl of a cycloheximide dilution in complete RPMI were added, resulting in a final concentration of 10 µg/ml. After 4, 8, 12, 16, and 20 h, the cells were lysed and protein expression of IKKϵ was determined by SDS-PAGE and immunoblotting.

### 2.15 Immunoprecipitation

For the analysis of TBK1-dependent IKKϵ ubiquitination, stable IKKϵ-GFP-expressing cells were generated by transducing IKKϵ-deficient THP1-Dual cells with IKKϵ-GFP-encoding lentivirus. Then, TBK1 protein expression was depleted by CRISPR-Cas9 genome editing. Ubiquitination of IKKϵ in TBK1 polyclonal KO cells was compared to IKKϵ-GFP-expressing WT cells by GFP-based immunoprecipitation (IP): Cells (1.5×10^7^ per condition, seeded at 10^6^ cells/ml in complete RPMI) were treated with the proteasome inhibitor MG-132 (10 µM, Cell Signaling Technologies) for 6 h to induce the accumulation of ubiquitinated proteins. Then, the cells were washed with PBS twice before they were lysed in TAP lysis buffer (100 mM NaCl, 50 mM Tris/HCl, 1.5 mM MgCl_2_, 5% (v/v) glycerol, 0.5% (v/v) IGEPAL CA-630, pH 7.5) supplemented with cOmplete Mini EDTA-free protease inhibitor (Roche), PhosSTOP phosphatase inhibitor cocktail (Roche), and the deubiquitinase inhibitor N-ethylmaleimide (10 mM) for 30 min on ice. After sonicating the samples to mechanically disrupt the nuclei (VialTweeter Sonication device, Hielscher Ultrasonics), lysates were cleared by centrifugation at 21,000×g for 20 min at 4°C. Then, the protein concentrations were determined using a Pierce BCA Protein Assay Kit (Thermo Fisher Scientific) according to the manufacturer’s instructions. 40 µl equilibrated GFP-trap magnetic agarose beads (Chromotek) were incubated with 1500 µg protein in 750 µl TAP lysis buffer supplemented with protease inhibitor, phosphatase inhibitor, and N-ethylmaleimide (10 mM) overnight on a tube rotator at 4°C. The next morning, beads were washed three times with TAP lysis buffer, followed by two washes with TAP wash buffer (100 mM NaCl, 50 mM Tris/HCl, 1.5 mM MgCl_2_, 5% (v/v) glycerol, pH 7.5). Then, the beads were incubated in 60 µl 2x Laemmli buffer for 10 min at 95°C, shaking at 350 rpm, to elute and denature the bound proteins. Input controls (2% of lysate used for IP) and eluates were analyzed by SDS-PAGE (30 µl sample per gel) and immunoblotting.

To detect non-covalent interactions of TBK1 with TANK, NAP1, SINTBAD, and IKKϵ, tag-based co-IP was performed. Flag-tagged TBK1 WT, TBK1 L693A, TBK1 K694E, or TBK1 L704A was stably expressed in TBK1-deficient THP1-Dual cells by lentiviral transduction followed by puromycin selection. Cells (1.5×10^7^) were washed with PBS twice and lysed in 700 µl TAP lysis buffer supplemented with protease and phosphatase inhibitor by incubation on ice for 30 min with regular vortexing. The lysates were subjected to sonication and cleared by centrifugation, as described above, before protein concentrations were determined by BCA assay. After equilibrating anti-Flag M2 magnetic agarose beads (50 µl per condition, Sigma-Aldrich) in TAP lysis buffer, 2000 µg protein diluted in 1000 µl TAP lysis buffer (supplemented with protease and phosphatase inhibitor) were added to the beads and incubated for 4 h at 4°C, shaking at 900 rpm. The beads were washed three times with TAP lysis buffer and twice with TAP wash buffer, before elution of the precipitated proteins was achieved by incubation with 3× Flag peptide (Bachem, 150 ng/µl in TAP wash buffer) for 30 min at 4°C, shaking at 700 rpm. After adding Laemmli buffer to a final volume of 80 µl, the input controls (2% of lysate used for IP) and eluates were heat-denatured at 95°C for 5 min, before they were analyzed by SDS-PAGE (40 µl sample per gel) and immunoblotting.

### 2.16 Statistical analysis

GraphPad Prism 7 was used to perform statistical analysis. Individual values are visualized as dots, whereas columns and error bars represent the mean and standard deviation (SD) of three or more independent experiments or donors, respectively. The statistical significance of differences between groups was determined using the appropriate statistical test, as described in the figure legends. * indicates a p-value ≤ 0.05, ** a p-value ≤ 0.01, *** a p-value ≤ 0.001, and **** a p-value ≤ 0.0001.

## 3 Results

### 3.1 TBK1 mutation but not depletion results in diminished RLR-MAVS and cGAS-STING signaling in human monocytic cells

**Figure 1:**
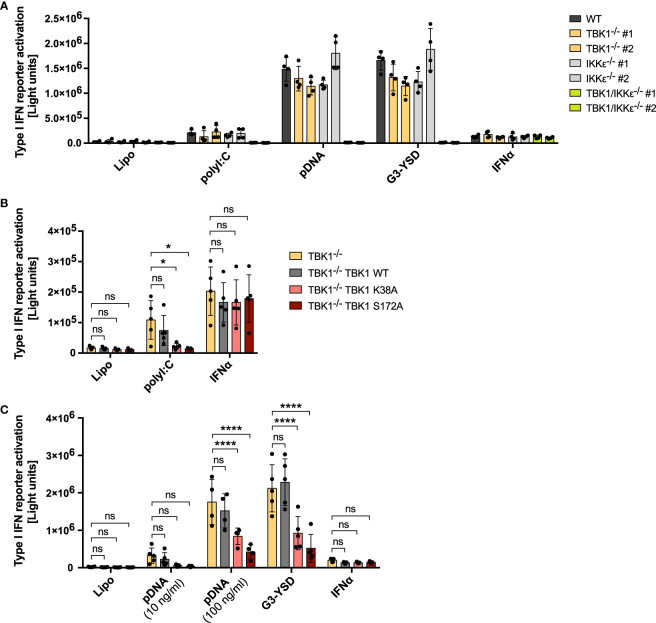
RLR and cGAS signaling in human monocytic cells is reduced in cells expressing kinase-deficient mutant TBK1 but not in TBK1-depleted cells. Type I IFN reporter gene expression in THP1 monocytes after stimulation for 16 h **(A, B)** or 24 h **(C)** with the RIG-I/MDA5 ligand polyI:C (100 ng/ml), the cGAS ligands plasmid DNA (pDNA, 100 ng/ml, unless indicated otherwise) or G3-YSD (500 ng/ml), IFNα (1000 U/ml), or with vehicle only (Lipo). Shown is the mean ± SD of n ≥ 4 independent experiments. Individual values are visualized as dots. **(B, C)** TBK1-deficient cells ectopically expressing Flag-tagged TBK1 WT, TBK1 K38A, or TBK1 S172A were compared to the TBK1-deficient parental cell line (clone #2) by two-way ANOVA followed by Dunnett’s multiple comparisons test (ns – not significant, * p ≤ 0.05, **** p ≤ 0.0001).

Although TBK1 is well-known for its essential function in innate immune responses in mice, loss of human TBK1 surprisingly is not associated with an increased susceptibility to severe infections (24). Based on the observation from this recent study, we aimed at further investigating the consequences of TBK1 deletion for antiviral defense of human monocytic cells, essential players in innate immune recognition and important inducers of type I IFN upon viral infection. We decided to study the type I IFN responses initiated by key sensors of viral nucleic acids: the DNA sensor cGAS and the RLRs RIG-I and MDA5. Although TBK1 is described as important kinase for IRF activation downstream of these receptors, its depletion in THP1 monocytes by CRISPR-Cas9 genome editing (**Supplementary Figure 1A**) did not result in a prominent reduction of type I IFN reporter activity after RIG-I and MDA5 activation with polyI:C and after cGAS activation with the short Y-form DNA G3-YSD (27) or plasmid DNA (pDNA) (**Figure 1A**). Since functional redundancies between TBK1 and IKKϵ have been suggested, we also analyzed IKKϵ-depleted cells and cells deficient for both kinases (**Supplementary Figure 1A**). While knockout (KO) of IKKϵ did not result in a pronounced reduction of type I IFN induction after activation of RLRs and cGAS, depletion of both TBK1 and IKKϵ (TBK1/IKKϵ KO) abrogated the type I IFN response induced by these receptors (**Figure 1A**), suggesting that IKKϵ can substitute the function of TBK1 in these pathways, and vice versa. In contrast, TBK1/IKKϵ KO did not affect IFNAR signaling induced by IFNα, as expected (**Figure 1A**). Notably, overexpression of IKKϵ in TBK1/IKKϵ KO cells completely rescued the cGAS-induced type I IFN response, demonstrating that IKKϵ is capable to induce type I IFN independently of TBK1 (**Supplementary Figure 1B, C**).

To understand why patients with TBK1 kinase mutation but not TBK1 deletion suffer from hyper-susceptibility to viral infections, we aimed at comparing the type I IFN response of TBK1-deficient with that of TBK1-mutated monocytic cells. Therefore, we transduced TBK1-deficient cells with lentiviral particles encoding the kinase-dead TBK1 mutants K38A or S172A, which are unable to phosphorylate target proteins, or TBK1 wildtype (WT) as a control, and assessed the capability of these cell lines to induce type I IFN in response to RLR or cGAS ligands. Strikingly, while expression of TBK1 WT did not affect the induction of type I IFN after stimulation with polyI:C, pDNA, or G3-YSD, TBK1 K38A or TBK1 S172A expression resulted in significantly impaired type I IFN responses (**Figure 1B, C**). In contrast, IFNAR signaling triggered by IFNα treatment was unaffected by the expression of these mutants (**Figure 1B, C**). Of note, TBK1 K38A or TBK1 S172A overexpression in WT cells with endogenous TBK1 was not associated with reduced type I IFN induction after RLR-MAVS and cGAS-STING activation (**Supplementary Figure 2**), excluding a general dominant-negative impact of these kinase-deficient mutants on these pathways.

**Figure 2:**
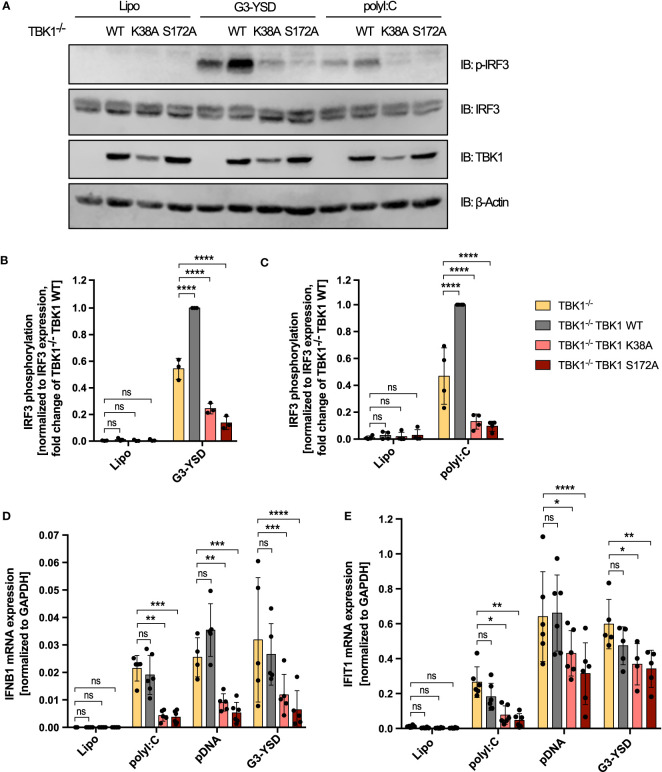
Decreased RLR- and cGAS-induced IRF3 phosphorylation and *IFNB1* expression in TBK1 mutant-expressing compared to TBK1-depleted cells. **(A)** Immunoblot (IB) analysis of TBK1-deficient THP1 cells (clone #2), untransduced or transduced with either Flag-tagged TBK1 WT, kinase-dead TBK1 K38A mutant, or kinase-dead TBK1 S172A mutant, stimulated for 3 h with the cGAS ligand G3-YSD (500 ng/ml), the RIG-I/MDA5 ligand polyI:C (100 ng/ml), or with vehicle only (Lipo). One representative blot of n = 3 (G3-YSD) or n = 4 (polyI:C) independent experiments is shown. **(B, C)** Quantification of IBs shown in **(A)** Levels of phosphorylated IRF3 after stimulation with G3-YSD **(B)** or polyI:C **(C)** normalized to total IRF3 expression levels are shown as the fold change of cells transduced with TBK1 WT (mean ± SD of n ≥ 3 independent experiments). **(D, E)** *IFNB1* **(D)** and *IFIT1* **(E)** mRNA levels of TBK1-deficient THP1 monocytes, untransduced or transduced with Flag-tagged TBK1 WT, TBK1 K38A, or TBK1 S172A, stimulated for 6 h with the RIG-I/MDA5 ligand polyI:C (100 ng/ml), the cGAS ligands pDNA (100 ng/ml) or G3-YSD (500 ng/ml), or vehicle only (Lipo). Shown is the mean ± SD of n ≥ 4 independent experiments. **(B–E)** Individual values are visualized as dots. TBK1-deficient cells ectopically expressing Flag-tagged TBK1 WT, TBK1 K38A, or TBK1 S172A were compared to the TBK1-deficient parental cell line (clone #2) by two-way ANOVA followed by Dunnett’s multiple comparisons test (ns – not significant, * p ≤ 0.05, ** p ≤ 0.01, *** p ≤ 0.001, **** p ≤ 0.0001).

Downstream of RLRs or cGAS, type I IFN is induced through activation of the transcription factors IRF3 and IRF7 (4). To understand whether TBK1 loss and TBK1 mutation also differentially affect transcription factor activation, we next analyzed IRF3 phosphorylation at S396 as a measure of its activation. Both cGAS activation by G3-YSD and RLR activation by polyI:C resulted in substantial induction of IRF3 phosphorylation (**Figure 2A**), as expected. As observed for type I IFN induction, expression of TBK1 K38A or TBK1 S172A significantly reduced IRF3 phosphorylation induced by G3-YSD or polyI:C compared to TBK1-deficient cells (**Figures 2A–C**). However, in contrast to type I IFN induction, IRF3 phosphorylation was more pronounced in cells expressing TBK1 WT than in TBK1-deficient cells (**Figures 2A–C**), indicating that IRF3 phosphorylation is more dependent on TBK1 than type I IFN expression. This suggests that type I IFN induction downstream of RLR or cGAS activation is not exclusively driven by IRF3 in THP1 monocytes but that other transcription factors, e.g. IRF7, play a role as well.

We additionally measured the cGAS- or RLR-triggered induction of *IFNB1* mRNA in THP1 monocytes. Expression of TBK1 K38A or TBK1 S172A resulted in a strong reduction of induced *IFNB1* compared to cells devoid of TBK1 (**Figure 2D**), and mRNA expression from the IFN-stimulated gene *IFIT1* was impaired by TBK1 mutant expression as well (**Figure 2E**). In contrast, expression from both genes was unaffected by TBK1 WT expression in TBK1-depleted cells (**Figures 2D, E**), corroborating the findings obtained from previous type I IFN reporter assays.

In summary, the data show that kinase activity-abolishing mutations but not depletion of TBK1 in human monocytic cells result in impaired RLR- and cGAS-induced type I IFN responses. The inhibited type I IFN production in monocytes correlates with the insufficient virus control observed in patients with mutation-induced loss of TBK1 kinase activity but not in individuals with complete absence of TBK1 expression (24, 25), suggesting that dysfunction in these PRR pathways contributes to the susceptibility of these patients to severe viral infection.

### 3.2 TBK1 controls the expression of its related kinase IKKϵ on a post-transcriptional level

**Figure 3:**
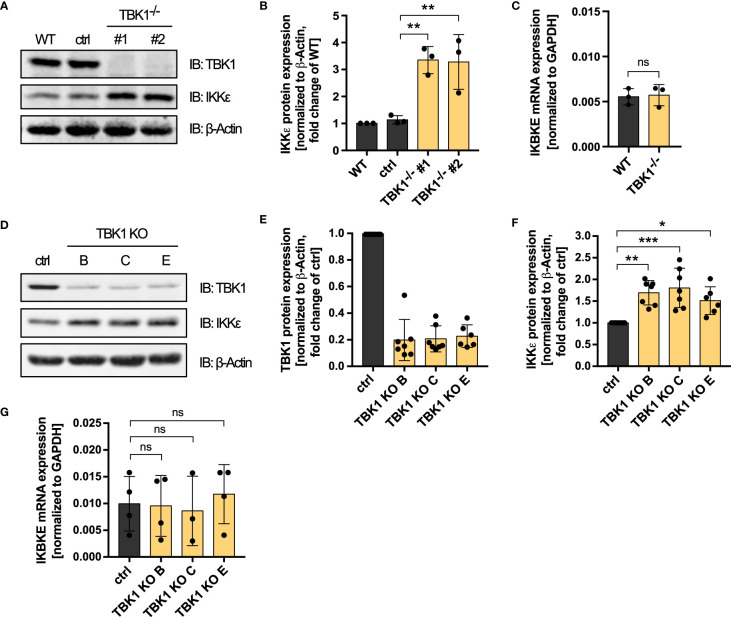
TBK1 diminishes the protein expression of IKKϵ but does not affect its mRNA levels. **(A)** Immunoblot (IB) analysis of THP1 wildtype (WT) cells, cells that have undergone the CRISPR-Cas9 genome editing procedure with a control crRNA (ctrl), and two TBK1-deficient monoclonal cell lines. One representative blot of n = 3 independent lysate preparations is shown. **(B)** Quantification of IBs shown in **(A)**. IKKϵ protein levels normalized to the expression of the housekeeping protein β-Actin are shown as the fold change of WT cells (mean ± SD of n = 3 independent lysate preparations). Individual values are visualized as dots. One-way ANOVA followed by Dunnett’s multiple comparisons test was performed to compare ctrl cells with TBK1-deficient cells (** p ≤ 0.01). **(C)** *IKBKE* mRNA levels from THP1 WT cells and TBK1-deficient cells, normalized to the mRNA expression of the housekeeping protein GAPDH, are shown as the mean ± SD of n = 3 independent RNA preparations. Individual values are visualized as dots. An unpaired t test was performed to compare WT with TBK1-deficient cells (ns – not significant). **(D)** IB analysis of primary human monocyte-derived macrophages subjected to CRISPR-Cas9 genome editing, either using a non-targeting control crRNA (ctrl) or TBK1 crRNAs B, C, or E (TBK1 KO). One representative blot from n = 7 independent experiments with different healthy donors is shown. **(E, F)** Quantification of IBs presented in **(D)**. TBK1 **(E)** and IKKϵ **(F)** protein expression levels normalized to the expression of the housekeeping protein β-Actin are shown as the fold change of ctrl cells (mean ± SD of n = 7 independent experiments with different healthy donors). Individual values are visualized as dots. **(G)** *IKBKE* mRNA levels from primary human monocyte-derived macrophages subjected to CRISPR-Cas9 genome editing, either using a non-targeting control crRNA (ctrl) or TBK1 crRNAs B, C, or E (TBK1 KO). *IKBKE* levels normalized to *GAPDH* expression are shown as the mean ± SD of n = 3–4 independent experiments with different healthy donors. Individual values are visualized as dots. **(F, G)** One-way ANOVA followed by Dunnett’s multiple comparisons test was performed to compare ctrl and TBK1 KO cells (ns – not significant, * p ≤ 0.05, ** p ≤ 0.01, *** p ≤ 0.001).

The function of TBK1 in activating IRF transcription factors downstream of RLRs and cGAS depends on its kinase activity. However, why expressed but inactive TBK1 inhibits type I IFN induction more than TBK1 loss has so far remained mechanistically unclear. The finding that TBK1 and IKKϵ have redundant functions in RLR and cGAS signaling in human monocytic cells (**Figure 1A**, **Supplementary Figures 1B, C**) prompted us to hypothesize that IKKϵ might play a role for the observed difference. When analyzing TBK1- and IKKϵ-deficient cells in the course of KO validation, we strikingly realized that TBK1-deficient cells exhibited enhanced protein expression levels of IKKϵ (**Supplementary Figure 1A**). To further examine this observation, we quantified IKKϵ expression of two TBK1-deficient monoclonal cell lines, WT cells, and control cells, which have gone through the CRISPR-Cas9 genome editing procedure with a non-targeting control crRNA. Lack of TBK1 expression was associated with a more than 3-fold induction of IKKϵ protein expression compared to control cells (**Figures 3A, B**), suggesting that a loss of TBK1 results in the upregulation of IKKϵ. While IKKϵ protein expression was strongly affected by the presence of TBK1, *IKBKE* mRNA levels were found unchanged between WT and TBK1-deficient cells (**Figure 3C**), pointing towards a post-transcriptional control of IKKϵ expression by TBK1.

In addition to the THP1 monocyte model, we investigated the influence of TBK1 depletion on IKKϵ protein expression in human primary monocyte-derived macrophages. Although TBK1 protein expression could be reduced by only approximately 80%, polyclonal TBK1 KO macrophages generated by CRISPR-Cas9 genome editing with either of three different TBK1-specific crRNAs exhibited a significant induction of IKKϵ protein expression compared to macrophages electroporated with a non-targeting control crRNA (**Figures 3D–F**). As in THP1 monocytes, *IKBKE* mRNA levels were unaffected by TBK1 KO in primary human monocyte-derived macrophages (**Figure 3G**), which indicates that the post-transcriptional regulation of IKKϵ is conserved among monocytic cells. In contrast to monocytes/macrophages, TBK1 depletion in HEK293FT cells did not result in increased protein expression of IKKϵ (**Supplementary Figure 3**), suggesting that the observed kinase cross-regulation might be a specific feature of myeloid cells, or of immune cells in general.

Overall, the data demonstrate that TBK1 diminishes the protein expression of its related kinase IKKϵ in both THP1 monocytes and primary human monocyte-derived macrophages. The resulting enhanced amounts of IKKϵ protein upon loss of TBK1 can substitute the function of TBK1 in IRF activation due to the functional redundancy of the kinases, which explains why RLR- and cGAS-induced type I IFN production is not substantially impaired in TBK1-deficient cells.

### 3.3 The kinase activity of TBK1 is not involved in repression of IKKϵ protein expression

**Figure 4:**
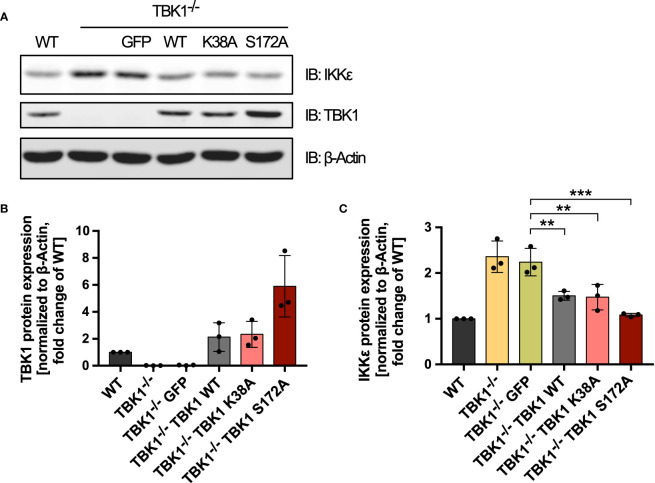
Kinase-dead TBK1 is still capable of reducing IKKϵ protein expression. **(A)** Immunoblot (IB) analysis of THP1 wildtype (WT) cells, TBK1-deficient cells (clone #2), and TBK1-deficient cells transduced with either GFP as a negative control or Flag-tagged TBK1 WT, kinase-dead TBK1 mutant K38A, or kinase-dead TBK1 mutant S172A. One representative blot of n = 3 independent lysate preparations is shown. **(B, C)** Quantification of IBs shown in **(A)**. TBK1 **(B)** and IKKϵ **(C)** protein expression normalized to the expression of the housekeeping protein β-Actin are shown as the fold change of WT cells (mean ± SD of n = 3 lysate preparations). Individual values are visualized as dots. **(C)** One-way ANOVA followed by Dunnett’s multiple comparisons test was used to compare IKKϵ expression of TBK1-transduced cell lines with that of GFP-expressing control cells (** p ≤ 0.01, *** p ≤ 0.001).

We next aimed at investigating whether the discovered TBK1-mediated control of IKKϵ protein expression is involved in the differential susceptibility to viral infection between patients with TBK1 deficiency and TBK1 mutation. We could show that IKKϵ plays an essential role in type I IFN induction downstream of RLRs and cGAS in TBK1-deficient cells (**Figure 1A**). Moreover, we observed that the expression of TBK1 mutants lacking kinase activity resulted in diminished type I IFN responses compared to TBK1-deficient cells (**Figures 1B, C**). We therefore hypothesized that the expression levels of IKKϵ might differ between these conditions. Indeed, not only re-expression of TBK1 WT but also expression of the TBK1 kinase-dead mutants K38A or S172A in TBK1-deficient THP1 monocytes resulted in significantly reduced levels of IKKϵ protein (**Figure 4**). These data suggest that TBK1 diminishes the protein expression of its related kinase IKKϵ independent of its kinase activity. Since kinase-dead TBK1 mutants are still capable to downregulate IKKϵ expression but cannot activate IRF3 and IRF7, cells expressing these mutants exhibit an overall reduced activity of IKK-related kinases compared to cells devoid of TBK1, in which IKKϵ protein expression is strongly enhanced. The reduced level of IRF-activating kinases is associated with a weaker type I IFN response downstream of RLR and cGAS activation, which likely explains the enhanced susceptibility of patients with TBK1 mutation to severe viral infection.

### 3.4 TBK1 enhances the degradation of IKKϵ independent of TBK1 ubiquitin ligase activity and K48-linked polyubiquitination

**Figure 5:**
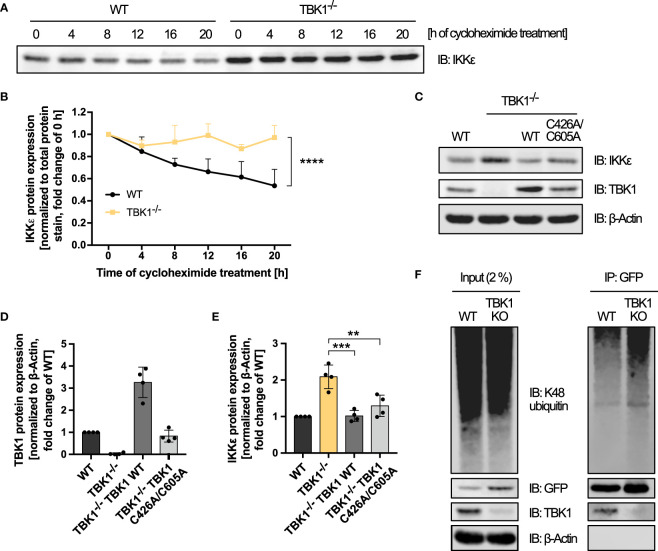
TBK1 controls the protein stability of IKKϵ independent of TBK1-intrinsic E3 ubiquitin ligase activity and K48 polyubiquitination. **(A)** Immunoblot (IB) analysis of IKKϵ expression in THP1 wildtype (WT) and TBK1-deficient cells (clone #2) treated with cycloheximide for the indicated time. One representative blot of n = 3 independent experiments is shown. **(B)** Quantification of the IKKϵ IBs from **(A)**. IKKϵ expression levels, normalized to total protein stain, are shown as the fold change of the respective untreated control (0 h; mean + SD of n = 3 independent experiments). Two-way ANOVA was performed to compare IKKϵ expression levels of WT and TBK1-deficient cells (**** p ≤ 0.0001). **(C)** IB analysis of the indicated proteins in WT cells, TBK1-deficient cells (clone #2), and TBK1-deficient cells transduced with either Flag-tagged TBK1 WT or TBK1 C426A/C605A mutant lacking E3 ubiquitin ligase activity. One representative blot of n = 4 independent lysate preparations is shown. **(D, E)** Quantification of IBs shown in **(C)**. TBK1 **(D)** and IKKϵ **(E)** protein expression levels normalized to the expression of the housekeeping protein β-Actin are shown as the fold change of WT cells (mean ± SD of n = 4 lysate preparations). Individual values are visualized as dots. **(E)** One-way ANOVA followed by Dunnett’s multiple comparisons test was used to compare IKKϵ expression of TBK1-transduced with that of untransduced TBK1-deficient cells (ns – not significant, ** p ≤ 0.01, *** p ≤ 0.001). **(F)** IKKϵ-deficient THP1 cells (clone #1) transduced with IKKϵ-GFP-encoding lentiviral particles (WT) were subjected to CRISPR-Cas9 genome editing with a TBK1-specific crRNA, resulting in TBK1 polyclonal knockout cells stably expressing IKKϵ-GFP (TBK1 KO). WT and TBK1 KO cells were treated for 6 h with the proteasome inhibitor MG-132 before whole-cell lysates were prepared and IKKϵ-GFP was immunoprecipitated (IP) with GFP nanobody-bound magnetic beads. Interacting or covalently bound proteins were detected by IB with the indicated antibodies. As input control, 2% of the lysate employed for immunoprecipitation was used. One representative blot of n = 3 independent experiments is shown.

We then further examined the mechanism of IKKϵ control by TBK1. Since the unaffected *IKBKE* mRNA expression suggested a post-transcriptional regulation, we investigated whether the protein stability of IKKϵ was influenced by TBK1. After blocking the synthesis of new proteins with the translation elongation inhibitor cycloheximide, protein degradation of IKKϵ in WT and TBK1-deficient THP1 monocytes was assessed by immunoblotting. Cycloheximide treatment of WT cells resulted in a decrease in IKKϵ protein expression over time, as expected (**Figures 5A, B**). In contrast, IKKϵ protein levels were not reduced throughout 20 h in TBK1-deficient cells (**Figures 5A, B**), indicating a much slower degradation rate of IKKϵ in absence of TBK1. Since cycloheximide treatment caused massive cell death in both WT and TBK1-deficient cells after more than 20 h incubation, the half-life of IKKϵ in presence and absence of TBK1 could not be determined with this method. However, statistical comparison of the two degradation curves revealed a highly significant difference in IKKϵ protein stability between TBK1-competent and -deficient cells (**Figure 5B**).

Selective protein degradation is commonly mediated by the ubiquitin-proteasome system. In a three-step enzymatic process that involves E3 ubiquitin ligases, ubiquitin moieties are transferred to lysine residues of target proteins to mark them for proteasomal degradation (28). To determine whether TBK1 reduces IKKϵ protein levels through proteasomal degradation, we examined IKKϵ protein expression after treatment of WT and TBK1-deficient cells with the proteasome inhibitor MG-132. While proteasome inhibition for 24 h resulted in massive cell death, precluding any analysis, a shorter treatment time of 6 h had no effect on IKKϵ protein levels (data not shown), which may result from the very long half-life of IKKϵ observed before (**Figures 5A, B**). Therefore, no reliable conclusion about the role of proteasomal degradation in TBK1-mediated control of IKKϵ expression could be drawn from these experiments.

Recently, TBK1 has been described to harbor a E3 ubiquitin ligase activity itself, requiring residues C426 and C605 (29). To investigate whether this intrinsic ubiquitin ligase activity of TBK1 is responsible for inducing IKKϵ protein degradation, we analyzed the expression of IKKϵ in TBK1-deficient cells expressing the ubiquitin ligase-deficient TBK1 mutant C426A/C605A. Similar to the kinase-dead mutants K38A and S172A, TBK1 C426A/C605A expression reduced the protein expression of IKKϵ compared to untransduced TBK1-deficient cells (**Figures 5C–E**). Thus, the putative C426/C605-dependent intrinsic E3 ubiquitin ligase activity of TBK1 is not required for the induction of IKKϵ degradation. Instead, another ubiquitin-conjugating enzyme might be recruited to TBK1 to target IKKϵ.

Among the different types of ubiquitin chains, K48-linked polyubiquitin is the most abundant proteasome-targeting signal (30). We therefore raised the question whether the presence of TBK1 enhances K48-linked ubiquitination of IKKϵ. To address this hypothesis, GFP-tagged IKKϵ was stably expressed in IKKϵ-deficient THP1 monocytes by lentiviral transduction to allow specific GFP-based immunoprecipitation. Polyclonal TBK1 KO in these cells resulted in a strong increase of IKKϵ-GFP expression (**Figure 5F**), proving that the regulation of IKKϵ degradation by TBK1 is maintained despite its ectopic expression as a GFP fusion protein. An efficient pulldown of IKKϵ-GFP was observed with GFP-binding magnetic beads in both TBK1-expressing and TBK1-depleted cells (**Figure 5F**). Furthermore, TBK1 co-immunoprecipitated with IKKϵ-GFP, demonstrating the interaction of the two kinases in non-activated myeloid cells (**Figure 5F**). Although pronounced K48-linked polyubiquitination of IKKϵ could be detected, it was not reduced by depletion of TBK1 (**Figure 5F**). Instead, the polyubiquitin signal was even enhanced for TBK1-deficient cells, which is most likely related to the increased amount of IKKϵ protein expressed in TBK1-deficient cells and immunoprecipitated from these cells (**Figure 5F**). Thus, the data suggest that TBK1 regulates IKKϵ degradation through a K48-polyubiquitin-independent mechanism.

### 3.5 Interaction of TBK1 with the scaffold protein TANK is required for the regulation of IKKϵ protein stability

**Figure 6:**
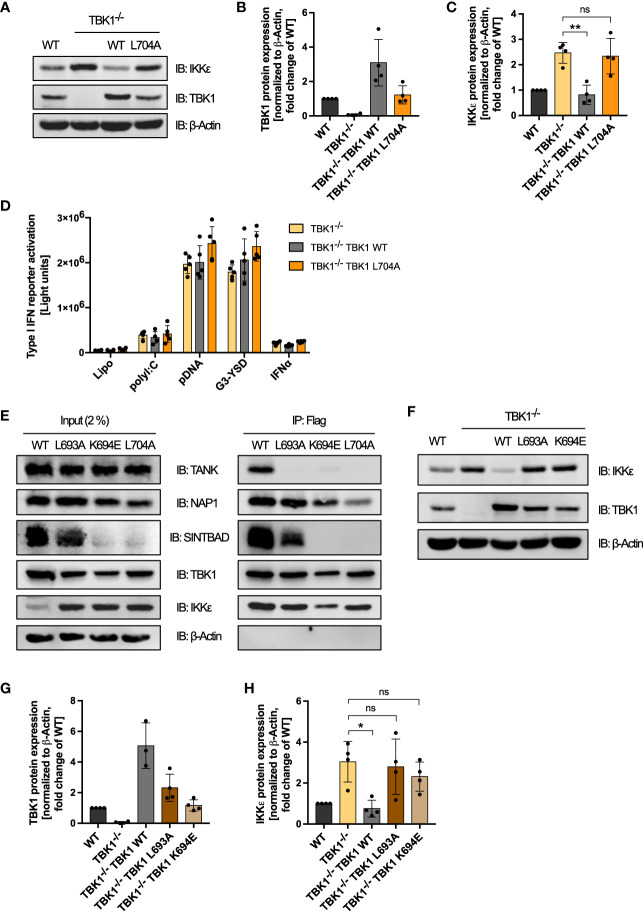
Interaction of TBK1 with the scaffold protein TANK is crucial for IKKϵ protein degradation. **(A)** Immunoblot (IB) analysis of WT cells, TBK1-deficient cells (clone #2), and TBK1-deficient cells transduced with either Flag-tagged TBK1 WT or TBK1 L704 mutant described to be unable to interact with the scaffold proteins TANK, NAP1, and SINTBAD. One representative blot of n = 4 independent lysate preparations is shown. **(B, C)** Quantification of IBs from **(A)**. TBK1 **(B)** and IKKϵ **(C)** protein expression levels normalized to the expression of the housekeeping protein β-Actin are shown as the fold change of WT cells (mean ± SD of n = 4 lysate preparations). Individual values are visualized as dots. **(C)** One-way ANOVA followed by Dunnett’s multiple comparisons test was used to compare IKKϵ expression of TBK1-transduced with that of untransduced TBK1-deficient cells (ns – not significant, ** p ≤ 0.01). **(D)** Type I IFN reporter gene expression in THP1 monocytes after stimulation for 24 h with the RIG-I/MDA5 ligand polyI:C (100 ng/ml), the cGAS ligands pDNA (100 ng/ml) or G3-YSD (500 ng/ml), IFNα (1000 U/ml), or vehicle only (Lipo). Shown is the mean ± SD of n = 5 independent experiments. Individual values are visualized as dots. **(E)** Whole-cell lysates of TBK1-deficient cells ectopically expressing Flag-tagged TBK1 WT, TBK1 L693A, TBK1 L694E, or TBK1 L704A were prepared and the TBK1 variants were immunoprecipitated (IP) with anti-Flag beads. Interacting proteins were detected by IB with the indicated antibodies. As input control, 2% of the lysate employed for immunoprecipitation was used. One representative blot of n = 3 independent experiments is shown. **(F)**. Immunoblot **(IB)** analysis of WT cells, TBK1-deficient cells (clone #2), and TBK1-deficient cells transduced with either Flag-tagged TBK1 WT, TBK1 L693A mutant that specifically lacks the ability to interact with TANK, or TBK1 K694E mutant unable to interact with the scaffold proteins TANK and SINTBAD. One representative blot of n = 4 independent lysate preparations is shown. **(G, H)** Quantification of IBs from **(F)** TBK1 **(G)** and IKKϵ **(H)** protein expression levels normalized to the expression of the housekeeping protein β-Actin are shown as the fold change of WT cells (mean ± SD of n = 4 lysate preparations). Individual values are visualized as dots. **(H)** One-way ANOVA followed by Dunnett’s multiple comparisons test was used to compare IKKϵ expression of TBK1-transduced with that of untransduced TBK1-deficient cells (ns – not significant, * p ≤ 0.05).

Since the enzymatic activities of TBK1 were found to be dispensable for its suppressive effect on IKKϵ protein levels, we hypothesized that instead the interaction of TBK1 with other proteins is essential. To investigate whether TBK1 induces the degradation of IKKϵ *via* interaction with one of its scaffold proteins, we expressed the TBK1 mutant L704A, described to be unable to interact with TANK, NAP1, and SINTBAD (16), in TBK1-deficient THP1 monocytes (**Figure 6A**). Strikingly, although the expression level of TBK1 L704A was comparable to TBK1 WT expression in WT cells, TBK1 L704A in contrast to TBK1 WT could not induce protein degradation of IKKϵ (**Figures 6A–C**). Thus, the interaction of TBK1 with its scaffold proteins seems to be crucial for the regulation of IKKϵ protein expression. In contrast to the kinase-dead mutants K38A and S172A, expression of TBK1 L704A in TBK1-deficient cells was not associated with a reduced type I IFN induction in response to RLR and cGAS ligands (**Figure 6D**). Since the interaction of TBK1 with scaffold proteins has been shown to be required for TBK1-mediated type I IFN induction (16), these data further demonstrate that the type I IFN response induced by RLRs and cGAS is determined by the level of IKKϵ expression in cells lacking active TBK1.

To further elucidate the determinants of TBK1-mediated IKKϵ degradation, we studied TBK1 mutants that have lost the ability to interact with individual scaffold proteins: While L693A mutation has been shown to selectively abrogate TANK binding, TBK1 K694E has been described to be unable to interact with TANK and SINTBAD and to only retain binding to NAP1 in HEK293 cells (16). To confirm that these findings also apply to TBK1 in monocytic cells, we expressed TBK1 WT, TBK1 L693A, TBK1 K694E, or TBK1 L704A in TBK1-deficient THP1 cells and analyzed the interaction of these TBK1 variants with the scaffold proteins TANK, NAP1, and SINTBAD by Flag-based co-immunoprecipitation. Unexpectedly, unlike described for HEK293 cells (16), L704A mutation decreased but not abolished NAP1 interaction in THP1 monocytes (**Figure 6E**). However, this mutant was unable to interact with TANK and SINTBAD (**Figure 6E**). Similar to L704A, K694E mutation abolished TBK1 interaction with TANK and SINTBAD, while NAP1 binding of this mutant was unaffected (**Figure 6E**). Of note, endogenous expression of SINTBAD was strongly reduced by expression of TBK1 K694E or TBK1 L704A (**Figure 6E**), suggesting that SINTBAD stability might be regulated by TBK1. Importantly, TBK1 L693A was unable to interact with TANK in human monocytic cells, while SINTBAD and NAP1 binding were maintained (**Figure 6E**), confirming the selective effect of this mutation that has been described in HEK293 cells (16).

Since we hypothesized that scaffold protein interaction of TBK1 is essential for complex formation with IKKϵ, we then compared the TBK1:IKKϵ interaction in cells expressing TBK1 WT, TBK1 L693A, TBK1 K694E, or TBK1 L704A. Strikingly, co-immunoprecipitation revealed that all TBK1 variants are capable of interacting with IKKϵ (**Figure 6E**). Thus, the scaffold proteins do not determine the association of TBK1 with IKKϵ but may rather link TBK1 to a mechanism that induces the degradation of IKKϵ.

To evaluate the involvement of the individual scaffold proteins in regulating IKKϵ protein stability, we next analyzed the effects of the TBK1 mutants L693A and K694E on IKKϵ expression. Although expressed to a similar level as TBK1 in WT cells, the TBK1 mutants L693A and K694E in contrast to TBK1 WT could not reduce IKKϵ protein levels in TBK1-deficient cells (**Figures 6F–H**). Since TBK1 L693A mutation selectively abolishes TANK binding but retains binding to NAP1 and SINTBAD, these data demonstrate that among the three scaffold proteins, TANK is required for TBK1-controlled IKKϵ degradation.

### 3.6 TBK1 depletion-triggered IKKϵ induction is an important backup mechanism to ensure unmitigated type I IFN responses during *Listeria monocytogenes* infection

**Figure 7:**
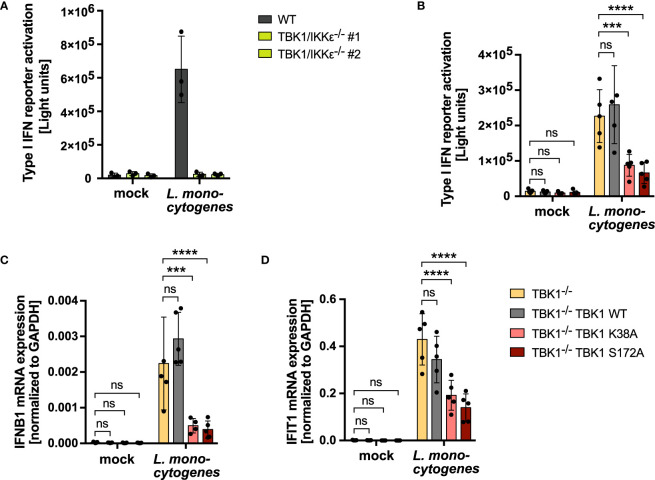
IKKϵ upregulation is crucial to compensate a loss of TBK1 during bacterial infection and ensure unmitigated type I IFN production. **(A)** Type I IFN reporter gene expression of *L. monocytogenes*-infected or mock-infected WT or TBK1/IKKϵ-deficient THP1 monocytes 24 h post infection. Shown is the mean ± SD of n = 3 independent experiments. Individual values are visualized as dots. **(B)** Type I IFN reporter gene expression of *L. monocytogenes*-infected or mock-infected TBK1-deficient THP1 monocytes (clone #2), untransduced or transduced with Flag-tagged TBK1 WT, TBK1 K38A, or TBK1 S172A, measured 24 h post infection. **(C, D)** *IFNB1* **(C)** and *IFIT1* **(D)** mRNA levels of *L. monocytogenes*-infected or mock-infected TBK1-deficient THP1 monocytes (clone #2), untransduced or transduced with Flag-tagged TBK1 WT, TBK1 K38A, or TBK1 S172A. Transcript levels 24 h post infection, normalized to *GAPDH* mRNA levels, are depicted. **(B–D)** Shown is the mean ± SD of n = 5 independent experiments. Individual values are visualized as dots. TBK1-deficient cells ectopically expressing Flag-tagged TBK1 WT, TBK1 K38A, or TBK1 S172A were compared to the TBK1-deficient parental cell line by two-way ANOVA followed by Dunnett’s multiple comparisons test (ns – not significant, *** p ≤ 0.001, **** p ≤ 0.0001).

Type I IFN production by innate immune cells is of imminent importance for the defense against invading pathogens. In consequence, viruses have evolved to antagonize type I IFN induction, such as by TBK1 degradation, which is described for SARS-CoV-2, HSV-1 and HIV-1 (18–21). Also bacteria have been found to subvert the production of type I IFNs *via* degradation of TBK1 (22). Based on our finding that RLR- and cGAS-induced type I IFN production in TBK1-deficient cells is strongly determined by the level of IKKϵ expression, we hypothesized that in case of TBK1 degradation during an infection, IKKϵ upregulation is highly relevant for efficient type I IFN responses and pathogen control. To address this hypothesis, we aimed at comparing infection-induced type I IFN production in 1) a condition of TBK1 deficiency in which IKKϵ is upregulated (TBK1-depleted cells) with 2) a condition of TBK1 deficiency in which IKKϵ is not upregulated (TBK1-depleted cells expressing inactive TBK1 K38A or S172A). We used *Listeria monocytogenes* (*L. monocytogenes*) as a model pathogen, as it is known to induce a type I IFN response *via* RLRs and cGAS/STING (31, 32). Infection of THP1 monocytes resulted in prominent type I IFN induction, as expected, which was found completely dependent on TBK1 and IKKϵ (**Figure 7A**). Strikingly, type I IFN induction after infection was strongly diminished in cells expressing the kinase-dead TBK1 mutants K38A or S172A, in which IKKϵ upregulation does not take place (**Figure 7B**). Moreover, mRNA levels of *IFNB1* and of the IFN-stimulated gene *IFIT1* were drastically reduced in these cells (**Figures 7C, D**). Overall, these data demonstrate that in conditions of TBK1 deficiency, IKKϵ upregulation is crucial to ensure efficient type I IFN production during infection.

## 4 Discussion

The IKK-related kinases TBK1 and IKKϵ are crucial components of type I IFN-inducing signaling pathways downstream of several PRRs, including RLRs and cGAS. Their function in these pathways is highly redundant, as demonstrated for human myeloid cells in this study. In consequence, loss of TBK1 can be functionally compensated by IKKϵ. Here, we have described an important regulatory mechanism contributing to this functional redundancy. We could show that TBK1 induces the constant degradation of IKKϵ under physiological conditions. In consequence, loss of TBK1 results in a prominent upregulation of IKKϵ protein expression, as demonstrated for THP1 cells as well as primary human monocyte-derived macrophages in this study. Since several pathogens, such as SARS-CoV-2, HIV-1, HSV-1, and *Shigella* species have been described to cause degradation of TBK1 (18–22), the discovered mechanism likely contributes to ensuring efficient type I IFN production by the host cell that facilitates antiviral or antibacterial defense even upon loss of TBK1.

Mechanistically, we have demonstrated that the catalytic activities of TBK1 are not involved in enhancing IKKϵ degradation: Kinase-dead TBK1 mutants were still capable of destabilizing IKKϵ protein, precluding that IKKϵ degradation is controlled by phosphorylation-dependent ubiquitination as it is known for IκBα in the course of NF-κB activation (33). Moreover, the recently described intrinsic E3 ubiquitin ligase activity of TBK1 (29) was found dispensable for reducing IKKϵ protein expression. Hence, TBK1 might induce IKKϵ degradation rather *via* the recruitment of co-factors. TBK1 is known to assemble different protein complexes depending on its interaction with the scaffold proteins TANK, NAP1, and SINTBAD (13). We could show that the capability of TBK1 to associate with these scaffolds is indispensable for its control of IKKϵ protein stability: Expression of TBK1 mutant L704A, which is unable to interact with TANK and SINTBAD and shows reduced binding of NAP1, did not reduce IKKϵ protein levels in human monocytic cells, although interaction with IKKϵ is retained, as demonstrated by co-immunoprecipitation. Among the three scaffold proteins, we identified TANK to be required for TBK1-directed IKKϵ degradation: Expression of the TBK1 mutant L693A, which specifically lacks the ability to interact with TANK but retains binding to NAP1 and SINTBAD, could not reduce protein expression levels of IKKϵ in TBK1-deficient cells. In light of our findings, we hypothesize that TBK1 *via* its scaffold TANK recruits a co-factor that directs the degradation of IKKϵ. To further elucidate the mechanism of IKKϵ destabilization, co-immunoprecipitation of TBK1 followed by mass spectrometry might be performed in future studies to identify potential co-factors. Evaluating the effects of CRISPR-Cas9-mediated candidate depletion on IKKϵ protein expression levels could then help to pinpoint the players involved in IKKϵ degradation.

Selective protein degradation is commonly mediated by the ubiquitin-proteasome system. Since within this system substrate specificity is mainly determined by E3 ubiquitin ligases (34), it is tempting to speculate that TBK1 recruits an E3 ubiquitin ligase to IKKϵ to mediate its ubiquitination and turnover. Several E3 ligases have been shown to control innate immune responses. While TRIM18-mediated ubiquitination stabilizes the TBK1 negative regulator PPM1A (35), other E3 ligases are described to directly induce the degradation of signaling-relevant proteins. For instance, TRIM29 has been shown to negatively regulate type I IFN responses by inducing the ubiquitination and degradation of MAVS and NEMO, key adaptor proteins in RLR signaling (36, 37). Of note, several E3 ligases such as DTX4, TRAIP, and TRIM27 have been demonstrated to target TBK1 itself for proteasomal degradation (38–40), rendering it likely that the TBK1-related kinase IKKϵ is regulated similarly through E3 ligases.

Among the different ubiquitin chain types, K48-linked polyubiquitin is the main proteasome-targeting signal (30). However, we could not detect enhanced K48-linked polyubiquitination of IKKϵ in TBK1-competent compared to TBK1-deficient cells, suggesting that this type of ubiquitination is not involved in the regulation of IKKϵ by TBK1. In addition to K48 linkages, K11- and K29-linked polyubiquitin chains as well as heterotypic chains consisting of different linkages have been shown to be involved in protein turnover *via* proteasomal degradation (30). Thus, other ubiquitin chain types might mediate the control of IKKϵ protein expression by TBK1. Alternatively, IKKϵ could be a target of ubiquitin-independent proteasomal degradation, which has been described for multiple substrates (41, 42).

In addition to the ubiquitin-proteasome system, the autophagy-lysosome pathway plays an essential role in protein degradation (43). Thus, it is also conceivable that the proteasome is not involved in TBK1-directed IKKϵ turnover. Importantly, TBK1 has already been implicated in regulating selective autophagy: It phosphorylates several autophagy modifiers and receptors such as optineurin, p62, and NDP52, and thereby initiates autophagy (44). Strikingly, selective autophagy has been demonstrated to play an important role in attenuating IFN signaling after cGAS activation: TBK1-mediated phosphorylation of p62 induces the autophagic degradation of STING, thereby limiting IRF3 activation and type I IFN production (45). In addition, IRF3 levels have been found to be reduced by NDP52-driven autophagy (46). It remains to be elucidated whether IKKϵ is also a target of TBK1-directed selective autophagy and which co-factors contribute to this process. In contrast to TBK1-driven autophagic degradation of STING, we have shown that IKKϵ destabilization does not depend on the kinase activity of TBK1. Thus, IKKϵ protein levels appear to be regulated *via* an alternative mechanism.

In this study, we found a conserved function of TBK1 in controlling IKKϵ expression in THP1 monocytes and primary monocyte-derived macrophages. However, TBK1 depletion in HEK293FT cells did not result in IKKϵ upregulation, arguing for a cell type-dependent mechanism. IKK-related kinases might be cross-regulated specifically in immune cells, or even exclusively in myeloid cells. Future studies should evaluate the effect of TBK1 depletion on IKKϵ expression in other cell types. Moreover, the determinants of such cell type specificities need to be identified. It is conceivable that TBK1 co-factors involved in IKKϵ degradation are not ubiquitously expressed, which could explain the observed differences.

Not only viruses but also bacteria are known to interfere with TBK1 function: For example, degradation of TBK1 by *Shigella* species has been described as a measure to dampen the host antibacterial response (22). Using *L. monocytogenes* as a bacterial model pathogen, which induces a type I IFN response that completely depends on TBK1 and IKKϵ in THP1 monocytes, we could show that IKKϵ regulation by TBK1 is essential for host responses to bacterial infection: Cells that lack active TBK1 but fail to upregulate IKKϵ showed a strongly diminished type I IFN and IFN-stimulated gene expression in response to bacterial infection compared to TBK1-deficient cells that are able to induce IKKϵ expression. These findings underline the importance of IKKϵ as an inducible back-up kinase that can substitute TBK1 functions in initiating type I IFN production in response to infections.

**Figure 8:**
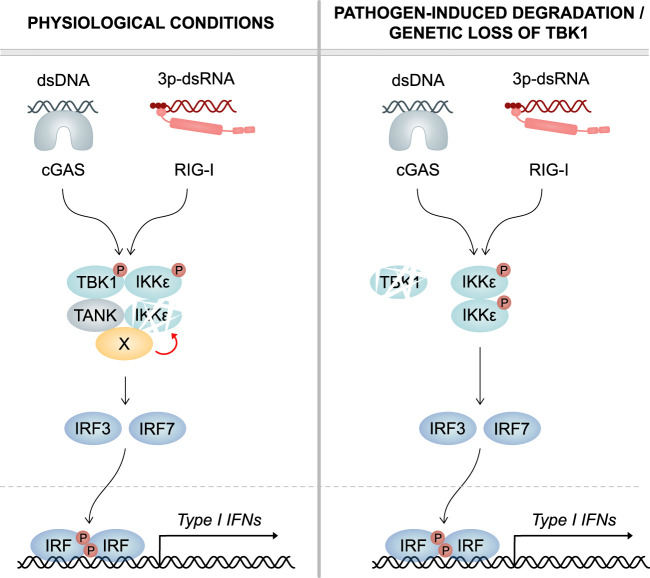
Model. *Left panel:* Under physiological conditions, TBK1 continuously induces the degradation of its related kinase IKKϵ, which has redundant functions in cGAS and RLR signaling. Mechanistically, TBK1 *via* its scaffold protein TANK recruits a co-factor (X) that destabilizes IKKϵ. Upon infection, both TBK1 and IKKϵ contribute to IRF activation and type I IFN production. *Right panel:* In case of pathogen-induced degradation or genetic loss of TBK1, IKKϵ protein stability is not reduced anymore. In consequence, IKKϵ protein levels are strongly enhanced. Due to the functional redundancy of the two kinases in IRF activation, increased amounts of IKKϵ protein can compensate for the lack of TBK1. This ensures unmitigated type I IFN responses and efficient clearance of infections.

In summary, based on the data presented here, we propose the following model: Dependent on its scaffold protein TANK, TBK1 recruits co-factors that accelerate the physiological turnover of IKKϵ in myeloid cells (**Figure 8**, left panel). In consequence, when TBK1 protein is lost, such as by viral or bacterial degradation, the protein stability of IKKϵ is enhanced, resulting in its accumulation in the cell. Due to the functional redundancy of TBK1 and IKKϵ in RLR- and cGAS-mediated type I IFN production, enhanced IKKϵ levels can compensate for the TBK1 deficiency (**Figure 8**, right panel). The here described regulatory mechanism ensures that type I IFN responses can take place efficiently, even in TBK1-depleted conditions.

Importantly, our study provides a mechanistic explanation for the previously unexplained observation that patients with point mutations in TBK1 that lead to kinase deficiency are highly susceptible to severe viral infection, while patients completely lacking TBK1 expression are not (24, 25): Kinase-dead TBK1 mutants are still capable of inducing the degradation of IKKϵ, as demonstrated for TBK1 K38A and TBK1 S172A in this study. In consequence, the overall amount of active IKK-related kinases is reduced. In contrast, loss of TBK1 results in an upregulation of IKKϵ expression, substituting the function of TBK1 in RLR- and cGAS-induced type I IFN responses. This compensation allows effective viral defense to take place and potentially prevents uncontrolled virus replication and severe disease.

The remarkable finding that TBK1 deficiency is better tolerated than TBK1 mutation in terms of immunocompetence opens up intriguing therapeutic opportunities: The development of intervention aiming at the degradation of mutant TBK1 would allow physiological IKKϵ upregulation to take place, leading to the restoration of an efficient type I IFN response. Moreover, the insights obtained in this study contribute substantially to the understanding of host cell mechanisms that counteract bacterial and viral antagonists and will potentially help to develop new strategies to fight infectious diseases.

## Data availability statement

The original contributions presented in the study are included in the article/**Supplementary Material**. Further inquiries can be directed to the corresponding author.

## Ethics statement

The studies involving human participants were reviewed and approved by Ethikkommission der Medizinischen Fakultät Bonn. The patients/participants provided their written informed consent to participate in this study.

## Author contributions

JW, CH and KC performed the experiments. JW and MS conceived the experiments. JW wrote the manuscript. GH and MS secured funding. All authors contributed to the article and approved the submitted version.
