# Supplementary material for: Increased IKKϵ protein stability ensures efficient type I interferon responses in conditions of TBK1 deficiency
Source: Front Immunol. 2023 Mar 3;14:1073608. doi: 10.3389/fimmu.2023.1073608 (PMC10020501; doi:10.3389/fimmu.2023.1073608)
Supplement: Supplementary file 1 [file DataSheet_1.pdf]

Supplementary Material

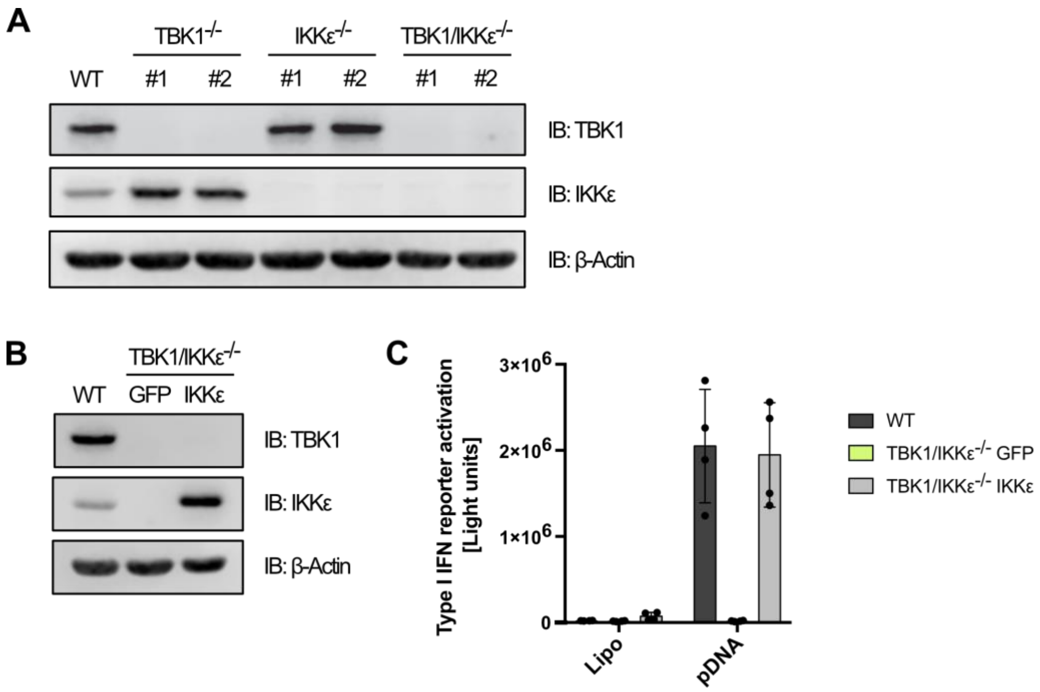

Supplementary Figure 1: Immunoblot analysis of TBK1-, IKKε- and TBK1/IKKε-deficient cell lines.

(A) Immunoblot (IB) analysis of THP1 WT cells and monoclonal cell lines lacking TBK1, IKKε, or TBK1 and IKKε (TBK1/IKKε$^{-/-}$). **(B)** Immunoblot (IB) analysis of THP1-Dual WT and TBK1/IKKε$^{-/-}$ cells, transduced with GFP as a negative control or Flag-tagged IKKε. **(A–B)** The housekeeping protein β-Actin serves as loading control. One representative blot of n = 3 independent lysate preparations is shown. **(C)** The cell lines shown in B were stimulated with the cGAS ligand pDNA (100 ng/ml) or vehicle only (Lipo). Type I IFN reporter activation 16 h post stimulation is shown as the mean ± SD of n = 4 independent experiments. Individual values are visualized as dots.

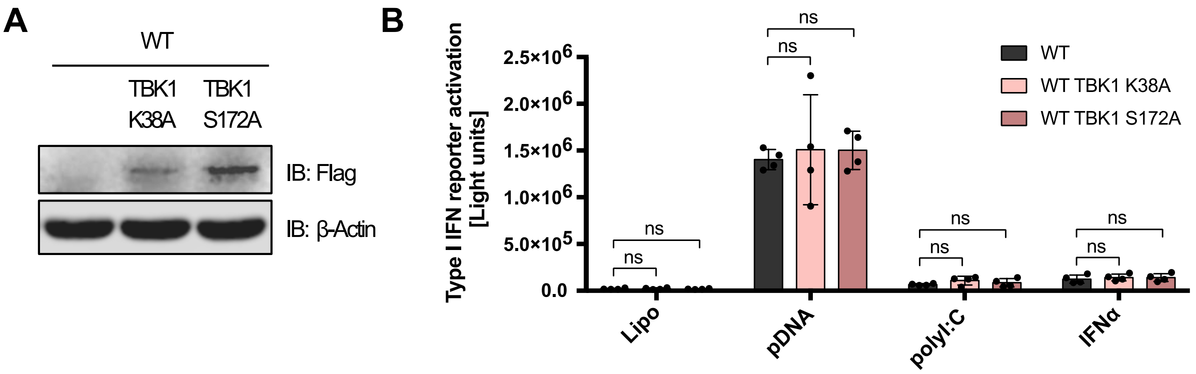

Supplementary Figure 2: Kinase-dead TBK1 mutants do not exhibit dominant-negative effects on cGAS and RLR signaling.

(A) Immunoblot (IB) analysis of WT cells, untransduced or transduced with lentiviral particles encoding Flag-tagged TBK1 K38A or TBK1 S172A. One representative blot of n = 3 independent lysate preparations is shown. **(B)** The cell lines shown in A were stimulated with ligands activating cGAS (pDNA, 100 ng/ml), RIG-I/MDA5 (polyI:C, 100 ng/ml), IFNAR (IFNα, 1000 U/ml), or vehicle only (Lipo). Type I IFN reporter activation 16 h post stimulation is shown as the mean ± SD of n = 4 independent experiments. Individual values are visualized as dots. Two-way ANOVA followed by Dunnett's multiple comparisons test was used to compare reporter activation of cells expressing TBK1 kinase-dead mutants with that of untransduced cells (ns – not significant).

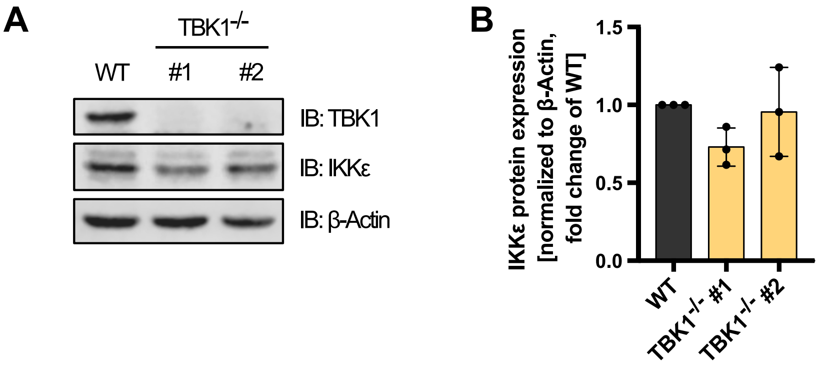

Supplementary Figure 3: TBK1 does not affect IKKε protein expression in HEK293FT cells.

(A) Immunoblot (IB) analysis of HEK293FT wildtype (WT) cells and two TBK1-deficient monoclonal cell lines. One representative blot of n = 3 independent lysate preparations is shown. **(B)** Quantification of IBs depicted in A. IKKε expression normalized to the expression of the housekeeping protein β-Actin is shown as the fold change of WT cells (mean ± SD of n = 3 independent lysate preparations). Individual values are visualized as dots.
